# Expression and Distribution of Ectonucleotidases in Mouse Urinary Bladder

**DOI:** 10.1371/journal.pone.0018704

**Published:** 2011-04-14

**Authors:** Weiqun Yu, Simon C. Robson, Warren G. Hill

**Affiliations:** 1 Renal Research Division, Beth Israel Deaconess Medical Center and Harvard Medical School, Boston, Massachusetts, United States of America; 2 Division of Gastroenterology, Beth Israel Deaconess Medical Center and Harvard Medical School, Boston, Massachusetts, United States of America; 3 Matrix Biology Division, Beth Israel Deaconess Medical Center and Harvard Medical School, Boston, Massachusetts, United States of America; Florida State University, United States of America

## Abstract

**Background:**

Normal urinary bladder function requires bidirectional molecular communication between urothelium, detrusor smooth muscle and sensory neurons and one of the key mediators involved in this intercellular signaling is ATP. Ectonucleotidases dephosphorylate nucleotides and thus regulate ligand exposure to P2X and P2Y purinergic receptors. Little is known about the role of these enzymes in mammalian bladder despite substantial literature linking bladder diseases to aberrant purinergic signaling. We therefore examined the expression and distribution of ectonucleotidases in the mouse bladder since mice offer the advantage of straightforward genetic modification for future studies.

**Principal Findings:**

RT-PCR demonstrated that eight members of the ectonucleoside triphosphate diphosphohydrolase (NTPD) family, as well as 5′-nucleotidase (NT5E) are expressed in mouse bladder. NTPD1, NTPD2, NTPD3, NTPD8 and NT5E all catalyze extracellular nucleotide dephosphorylation and in concert achieve stepwise conversion of extracellular ATP to adenosine. Immunofluorescent localization with confocal microscopy revealed NTPD1 in endothelium of blood vessels in the lamina propria and in detrusor smooth muscle cells, while NTPD2 was expressed in cells localized to a region of the lamina propria adjacent to detrusor and surrounding muscle bundles in the detrusor. NTPD3 was urothelial-specific, occurring on membranes of intermediate and basal epithelial cells but did not appear to be present in umbrella cells. Immunoblotting confirmed NTPD8 protein in bladder and immunofluorescence suggested a primary localization to the urothelium. NT5E was present exclusively in detrusor smooth muscle in a pattern complementary with that of NTPD1 suggesting a mechanism for providing adenosine to P1 receptors on the surface of myocytes.

**Conclusions:**

Ectonucleotidases exhibit highly cell-specific expression patterns in bladder and therefore likely act in a coordinated manner to regulate ligand availability to purinergic receptors. This is the first study to determine the expression and location of ectonucleotidases within the mammalian urinary bladder.

## Introduction

ATP is increasingly recognized as an important signaling mediator in the urinary bladder and is secreted both by the bladder epithelium or urothelium [1]–[3] and by neurons. Urothelium releases ATP both lumenally into the urine space and serosally. The mechanism underlying this release is not well understood but kinetic studies have shown that ATP secretion is markedly stimulated by stretch, indicating mechanically sensitive signaling pathways in response to bladder filling [2], [4]. ATP released lumenally from umbrella cells is thought to play a role in autocrine signaling while release from the serosal surfaces permits interaction with stromal elements including afferent neurons and possibly the detrusor as well [2], [5]. ATP is also released along with norepinephrine by postganglionic parasympathetic nerves that innervate the bladder smooth muscle resulting in a biphasic mechanical response that consists of an initial rapid twitch, followed by a sustained contraction [6].

Upon release, ATP can bind to purinergic receptors of the P2X and P2Y families and initiate ion transport or G-protein-coupled receptor signaling, respectively. P2X receptors, P2X_1_, P2X_2_, P2X_3_, P2X_5_, P2X_6_ and P2X_7_ are differentially expressed throughout the bladder [7] and loss of P2X_3_ from afferent nerve fibers in a knockout mouse was shown to alter voiding behavior by shifting the micturition reflex to greater fill volumes [8]. P2Y_2_ and P2Y_4_ also appear to be expressed [9] indicating a diverse repertoire of purinergic responsive receptors throughout all tissue elements of the bladder. Furthermore, abnormalities in ATP release and in purinergic receptor expression have been noted in numerous studies of human bladder disease as well as in animal models of bladder pathology. These include interstitial cystitis [10]–[12], urinary urgency and incontinence [13], bladder inflammation [14], spinal cord injury-induced bladder dysfunction [15], detrusor overactivity [16]–[17] and outlet obstruction [18]–[20].

While much research has focused on P1 and P2 receptors, purinergic signaling is also critically regulated by ectonucleotidases, which degrade ATP and UTP to their respective nucleosides. These enzymes act to limit, both temporally and quantitatively, the exposure of P2 receptors to their ligands [21]. They also preclude desensitization responses resulting from overstimulation. Furthermore, stepwise conversion creates potent metabolites, like ADP and adenosine, which may then continue to act through other receptors with different affinities and locations [22]. There are four main families of ectonucleotidases; NTPDs (ectonucleoside triphosphate diphosphohydrolases), NPPs (nucleotide pyrophosphatase/phosphodiesterases), alkaline phosphatases and ecto-5′-nucleotidase (NT5E). The families differ primarily in their substrate specificities, with NTPDs highly specific for ATP/UTP/ADP/UDP [21] while NPPs [23]–[24] catalyze phosphohydrolysis on a broader range of substrates including lysophospholipids and choline phosphate esters [22], [25]–[27]. Alkaline phosphatases are even more promiscuous with broad substrate specificities that overlap with those of the NPPs. Dysregulation of nucleotide metabolism and alterations to the activities of ectonucleotidases has been shown convincingly in many pathological conditions including diabetes, hypertension, acute stroke, chronic renal failure, cancer, myocardial infarction, leukemia and epilepsy [21], [28]–[29].

The presence of ectonucleotidases in bladder has not been studied systematically; however their existence was inferred, since the half-life of ATP is very different depending on which side of the urothelium it is released from. In Ussing chamber studies, Lewis and Lewis showed that both constitutive and stretch-induced ATP release from the luminal surface of rabbit bladders increase ATP concentration in a linear fashion with continuous accumulation, whereas serosal ATP rises and then plateaus – the kinetics of which are consistent with its initial release and then subsequent consumption [2].

Our long term goal is to develop an in-depth understanding of the regulation of purinergic signaling in bladder and its importance to normal and abnormal bladder function. Since secreted nucleotides are potent stimulators which may exert both autocrine and paracrine effects, our focus in these experiments was to determine the expression and location of cell-surface ecto-hydrolytic members of the NTPD family (NTPD1, -2, -3, and -8). Furthermore, as this group is not capable of completing the final phosphohydrolysis step which results in production of adenosine – another important signaling molecule, we also characterized tissue distribution of NT5E in bladder. Our findings suggest specific and synergistic mechanisms for the control of nucleotide availability throughout the stratified layers of the bladder.

## Materials and Methods

### Animals

Mice used in this study were C57BL/6J mice (19–21 g) from Charles River Laboratories (Wilmington, MA). Mice were euthanized by inhalation of 100% CO_2_. After euthanasia and thoracotomy, the bladders were rapidly excised and processed as described below. All animal studies were carried out with the approval of the Beth Israel Deaconess Medical Center Institutional Animal Care and Use Committee (Protocol #051-2009).

### Antibodies and labeled probes used for immunofluorescence (IF) and immunoblotting (IB)

Affinity-purified monoclonal rat anti-NTPD1 antibody (IF), affinity-purified polyclonal sheep anti-NTPD2 antibody (IF/IB), affinity-purified polyclonal sheep anti-NTPD3 antibody (IB) and affinity-purified monoclonal rat anti-NT5E antibody (IB) were purchased from R&D systems (Minneapolis, MN). Affinity-purified polyclonal rabbit anti-NTPD8 (IF/IB) was purchased from Sigma (St. Louis, MO). Affinity-purified polyclonal goat anti-NTPD8 antibody (IF) and anti-aquaporin 3 (IF) antibodies were purchased from Santa Cruz Biotechnology, Inc. (Santa Cruz, CA). Affinity purified goat polyclonal anti-CGRP antibody was purchased from ABCAM Inc. (Cambridge, MA). The following antibodies were from SCR's laboratory: affinity-purified polyclonal rabbit anti-NTPD1 antibody (IB), affinity-purified polyclonal rabbit anti-NTPD2 antibody (IF/IB); affinity-purified polyclonal rat anti-NT5E antibody (IF). Antibodies to FSP1 and αSMA were kind gifts from the laboratory of Dr. Raghu Kalluri (Beth Israel Deaconess Medical Center). Secondary donkey anti-rabbit/goat/rat antibodies conjugated to Alexa 488 or horseradish peroxidase (HRP), and Topro-3 and rhodamine-phalloidin were purchased from Invitrogen-Molecular Probes (Carlsbad, CA).

### RT-PCR analysis

Whole bladder RNA was extracted by a QIAGEN RNeasy Mini kit (Valencia, CA). RNA samples were treated with DNase I to remove potential genomic DNA contamination, and control reactions were performed in the absence of reverse transcriptase or in the presence of a control primer pair. Reverse transcription was carried out according to instructions for RETROscript (Ambion, Austin, TX). Primers were designed using Primer3 software (http://frodo.wi.mit.edu/primer3/) and standard PCR conditions were as follows: 95°C/10 min then 35 cycles of 95°/30 s; 50–60°/30 s; 72°/60 s followed by 72°/10 min. Optimal annealing temperature was experimentally determined. Primer pairs for each NTPD isoform are shown in Table 1. PCR products were run on a 2.0% agarose gel containing ethidium bromide to visualize the PCR products. Products were compared to a 100-bp ladder (PGC Scientific, Frederick, MD), which was used to estimate the size of the reaction products.

**Table 1:** Primers used for PCR of Nucleotidase Enzymes.

Enzyme	Sequence of Primers (5′-3′)	Expected Product size (bp)
NTPD1	TTTAGCGTTTTGTGTGGTTTTATATGTT	444
	CTGCCAAGTTCTTGGTAATAGAATGTTA	
NTPD2	AACCAGTCCATCTGAAGATCCAGATAAT	410
	AGTAGAAAGCAGAAAAGGCTATGAAGTT	
NTPD3	TATTTTTATTTTGCAGCGTGTATTTGTT	479
	CATAACTTTTATGTCTATGGGCATTTTG	
NTPD4	GCAGGAAGAAGTAGCTAAAAACCTGTTA	432
	CAGTAGTAGAACTCAGAGAAGCCGTAGA	
NTPD5	AAATCCTCAACCTTTTTAACTTTTCTCA	570
	ACAGTTCAGTTTTATTTATGGCTCCTCT	
NTPD6	CTAAGCAACACATTCCTTATGATTTCTG	599
	GACTTCTGATACTCTACTGGCACACAG	
NTPD7	GTGTGTTGAGAGATCTAGTCAGAAGTCC	556
	TAAATACTAAAACAGATGCAGCGAAAAC	
NTPD8	GGACTAGGTAGAAACCAAGCTGAAGTAG	506
	CAACTTGATCTATATTCTGGGATTGATG	
NT5E	GCTAGATATCAAGACTCACACACACAAA	526
	CACAGGAGTTAATAAGAACCAGTGTTGT	

### Western blot analysis of ectonucleotidases in bladder

Excised bladders were put in 1 ml ice cold radio-immunoprecipitation assay buffer (RIPA; 50 mM Tris pH 8.0, 150 mM NaCl, 1% v/v NP-40, 0.5% w/v deoxycholic acid, 0.1% w/v SDS) containing Complete Mini Protease Inhibitor Cocktail tablets (Roche, Germany). Proteins were resolved by SDS-PAGE and transferred to Immun-Blot PVDF membrane (BioRad Laboratories, Hercules, CA), and the blots were probed with specific antibodies as described earlier, followed by the appropriate species-specific secondary antibodies conjugated to HRP. Bands were detected using ECL Plus Western Blotting reagents (GE Healthcare, Piscataway, NJ) and CL-X Posure film (Thermo Scientific, Rockford, Il). The film was developed, scanned and images were contrast corrected with Photoshop (San Jose, CA), before importing into Adobe Illustrator CS3 (San Jose, CA).

### Immunofluorescence analysis of ectonucleotidases in bladder

Excised bladders were fixed in 4% (w/v) paraformaldehyde dissolved in 100 mM sodium cacodylate (pH 7.4) buffer for 2 h at room temperature. In some cases, tissue was fixed by 100% methanol (4°C) for 5 min. Fixed tissue was cut into small pieces with a razor blade, cryoprotected, frozen, sectioned (5 µm), and incubated with primary antibodies (1∶100 –1∶500 dilution) for 2 h at room temperature as described previously [30]. After washing away unbound primary antibody, the sections were incubated with a mixture of Alexa 488-conjugated secondary antibody (diluted 1∶100), rhodamine-phalloidin (1∶50), and Topro-3 (1∶1,000). The sections were washed with PBS, postfixed with 4% (wt/vol) paraformaldehyde, and mounted under coverslips with *p*-diaminobenzidine-containing mounting medium. All immunofluorescent localization data shown are representative images of staining performed on at least three individual bladders. As bladder is a highly distensible tissue, bladder sections prepared from individual animals varied greatly in tissue shape, folding and thickness, making quantitation with image analysis problematic. However, the clear architecture of the bladder, with readily defined layers and of structures within layers e.g. blood vessels, allowed us to assess the reproducibility of cellular expression with confidence. There was little variability noted between expression patterns from animal to animal.

### Scanning laser confocal analysis of fluorescently labeled cells

Imaging was performed on a Zeiss LSM-510 confocal microscope equipped with argon and green and red helium-neon lasers (Thornwood, NY). Images were acquired by sequential scanning with a 63X (1.4 numerical aperture) planapochromat oil objective and the appropriate filter combinations. Serial (*z*) sections were captured with a 0.25 µm step size. The images (512 & 512 pixels) were saved as TIFF files. Serial sections were projected into one image using LSM-510 software. The contrast level of the final images was adjusted in Adobe Photoshop, and the images were imported into Adobe Illustrator CS3.

## Results

**Figure 1:**
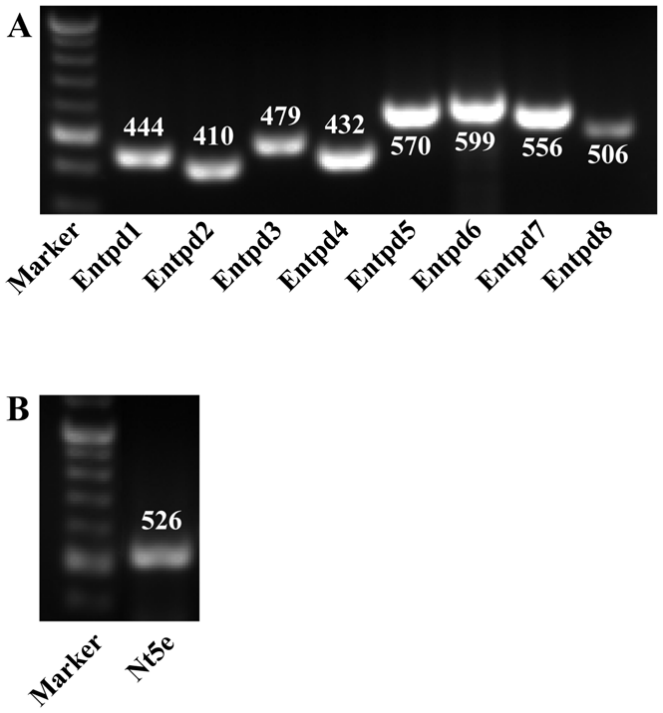
Expression of ectonucleotidases in mouse bladder by RT-PCR. Expression of ectonucleotidases was detected by RT-PCR from total RNA isolated from mouse bladder. Specific primers for each enzyme are given in Table 1. RT-PCR reaction products were resolved on 2% agrose gels and visualized by staining with ethidium bromide. Numbers above or beneath the DNA bands are expected product sizes (in bp). A) ENTPD family, B) NT5E.

Expression of all eight members of the NTPDase family and 5′-nucleotidase (NT5E) were examined in whole bladder extracts (Fig. 1). A broad expression profile at the level of mRNA for all of these enzymes was observed suggesting the presence of complex cellular and tissue regulation of nucleotide availability as well as a substrate scavenging capability.

**Figure 2:**
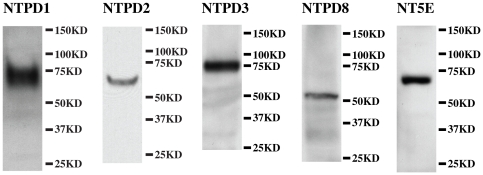
Western blotting for four ectoenzymes of the NTPD family and NT5E. Lysates of mouse bladder (25 µg protein/lane) were resolved by SDS-PAGE, and Western blots were probed with antibodies specific for five ectonucleotidases. Protein bands of the correct molecular weight were detected for each enzyme.

Immunoblotting of whole bladder lysates for NTPD1, -2, -3, -8 and NT5E demonstrated that all five proteins were detected at or near their predicted molecular weights of 57 kDa, 54 kDa, 59 kDa, 54 kDa and 64 kDa respectively (Fig. 2). NTPD1 and NTPD3 migrate somewhat higher than predicted and we attribute this to glycosylation or other post-translational modifications. Immunoblotting confirmed that these antibodies exhibited high specificity for the target antigens with little cross-reactivity to other proteins. Variability in expression levels between animals was assessed by running triplicate bladders (from three individuals) and quantitating band density for all five enzymes. When normalized to β actin staining, the % coefficient of variation (standard deviation/mean*100) was less than 15% for all (data not shown).

**Figure 3:**
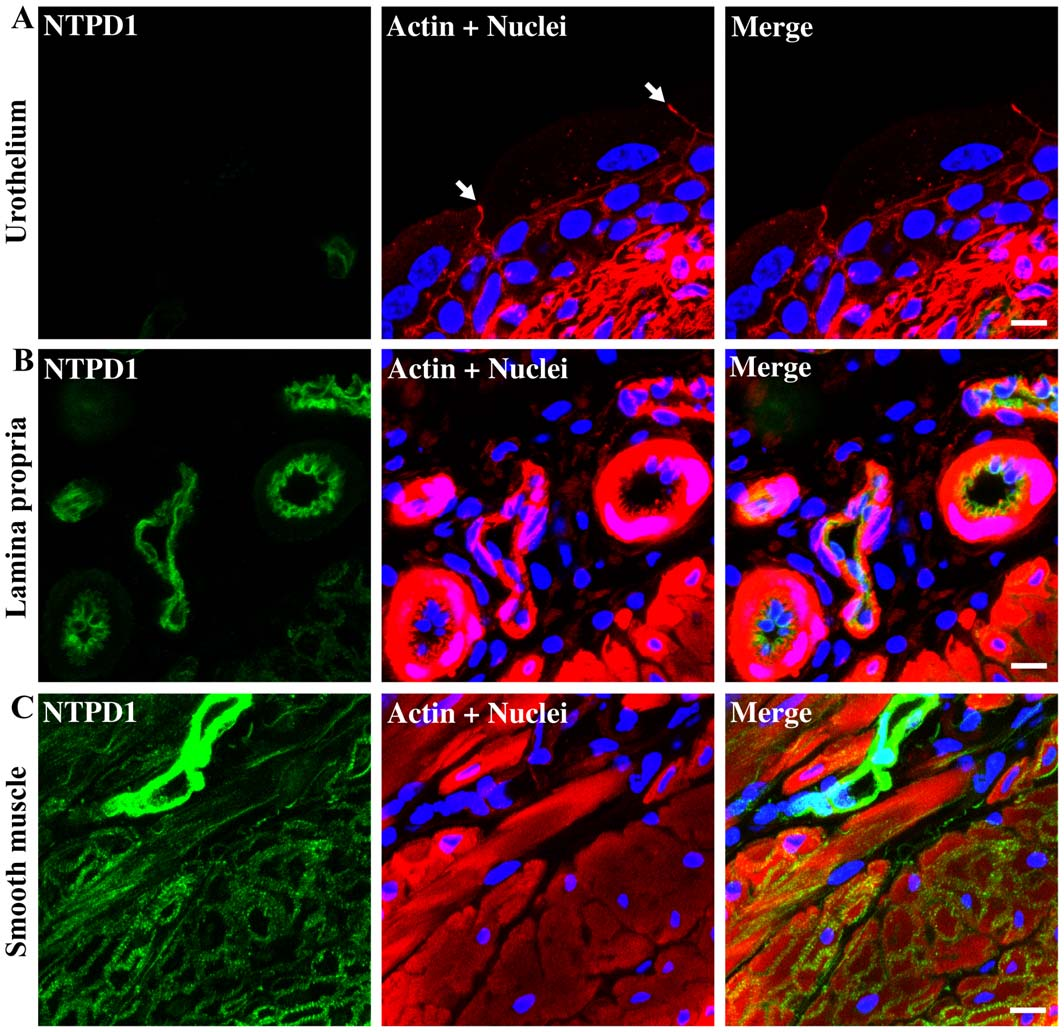
Immunolocalization of NTPD1 in different regions of the bladder. Cryosections of mouse bladders were labeled with antibodies to NTPD1 (green), rhodamine phalloidin to label the actin cytoskeleton (red) and Topro-3 to label nuclei (blue). Color merged panels are shown on the right. A) NTPD1 staining at the level of the urothelium; white arrows indicate tight junctions between superficial umbrella cells, B) NTPD1 staining at the level of the lamina propria, C) NTPD1 staining at the level of the detrusor smooth muscle. White scale bars  = 10 µm.

**Figure 4:**
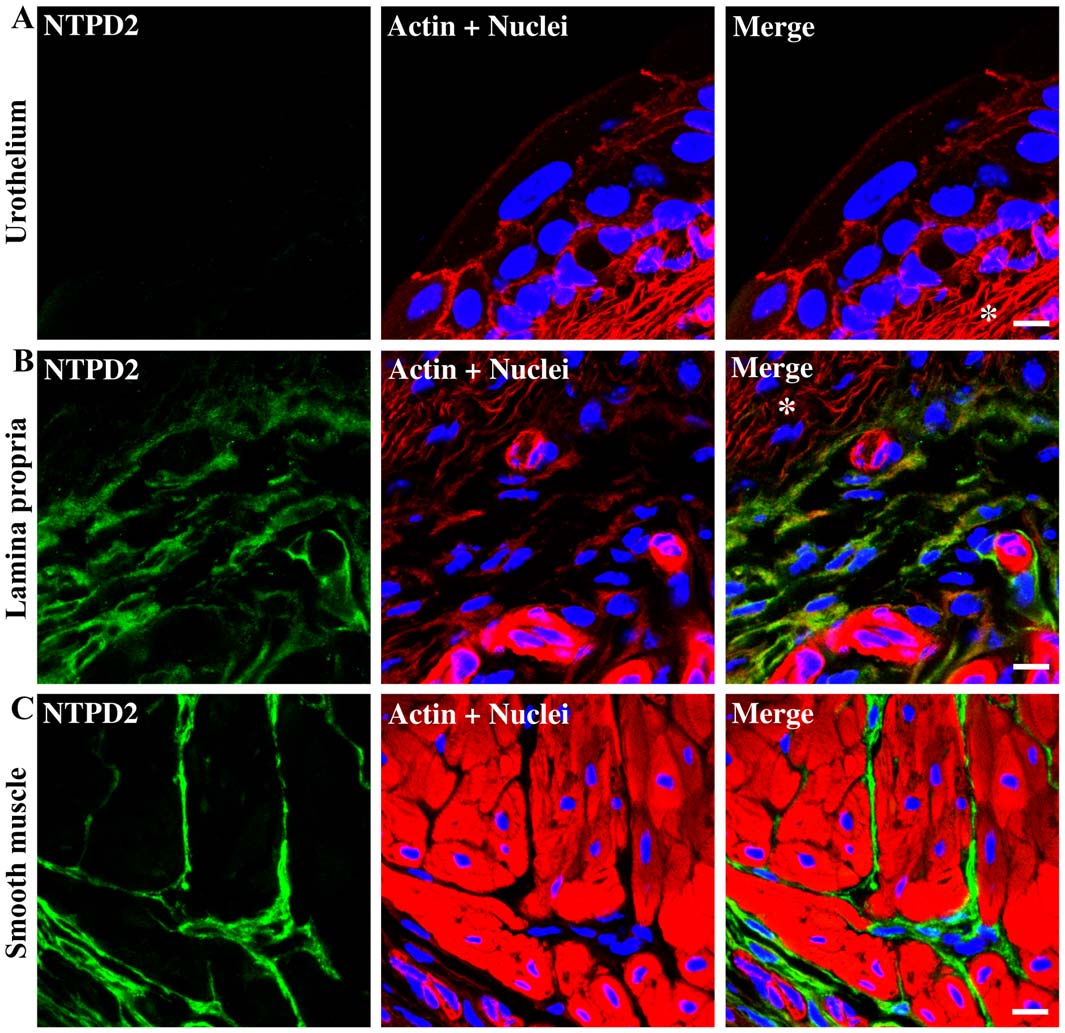
Immunolocalization of NTPD2 in different regions of the bladder. Cryosections of mouse bladders were labeled with antibodies to NTPD2 (green), rhodamine phalloidin to label the actin cytoskeleton (red) and Topro-3 to label nuclei (blue). Color merged panels are shown on the right. A) NTPD2 staining at the level of the urothelium, B) NTPD2 staining at the level of the lamina propria; white asterisks (*) indicate areas positive for actin but negative for NTPD2 in the lamina propria. C) NTPD2 staining at the level of the detrusor smooth muscle. White scale bars  = 10 µm.

Immunostaining of frozen bladder sections was performed for all five proteins of interest. By counterstaining actin with rhodamine-phalloidin (shown as red staining in middle panels, Figs. 3, 4, 6, 7, 8) we are able to clearly identify cell layers and tissue boundaries throughout the bladder. Confocal immunofluorescent laser scanning microscopy revealed that NTPD1 is expressed at high levels in endothelium of vascular elements occurring prominently within the lamina propria and is also present throughout the detrusor smooth muscle (Fig. 3b and 3c). There was no evidence for NTPD1 in the urothelium which is typically three cell layers deep (Fig. 3a). Merged panels on the right show that the protein is in or near plasma membranes as expected.

**Figure 5:**
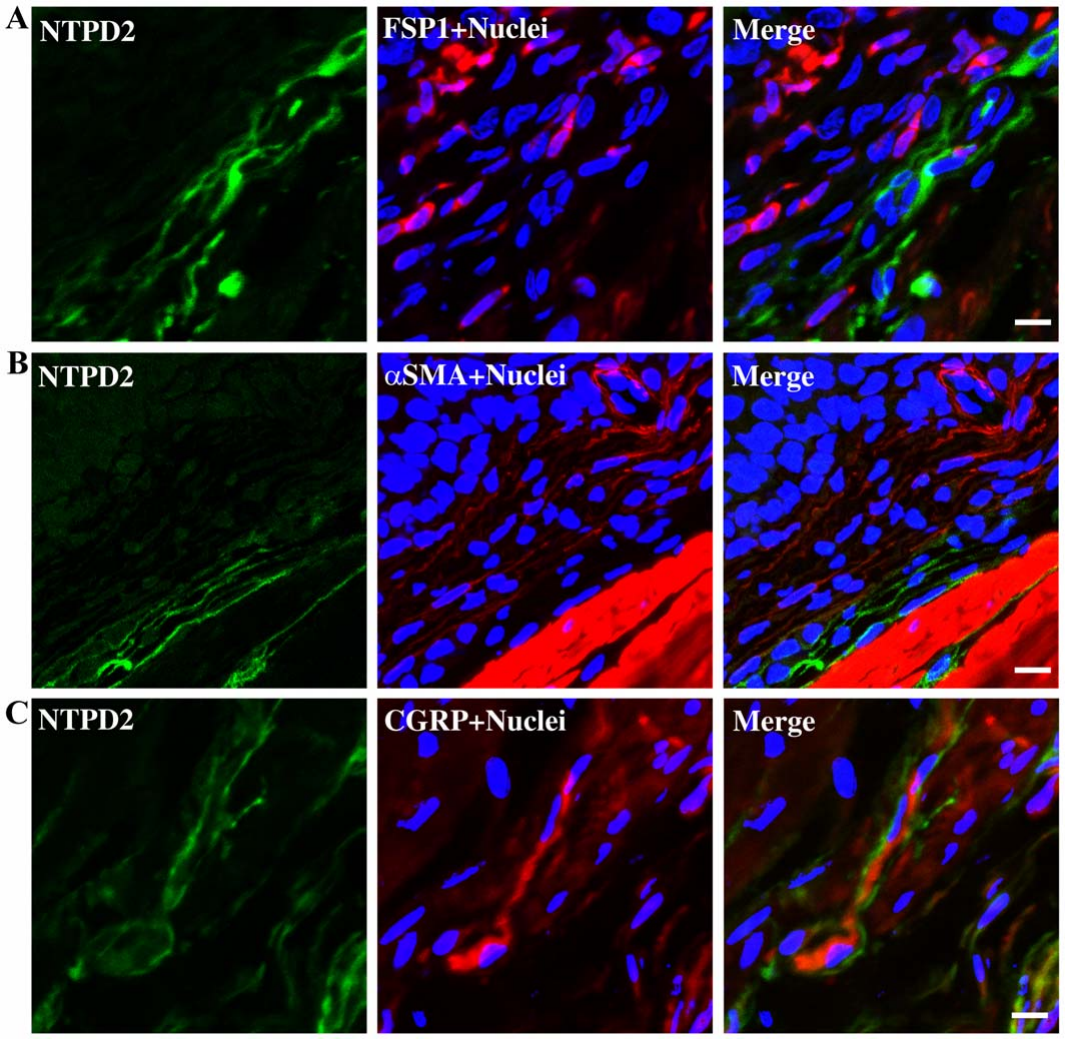
NTPD2 does not colocalize with fibroblasts, myofibroblasts or neurons. Cryosections of mouse bladders were coimmunostained with NTPD2 (green) and antibodies to marker proteins for A) fibroblasts – FSP1 (red), B) myofibroblasts – αSMA (red) and C) afferent nerves – CGRP (red). Nuclei are blue and color merged panels are shown on the right. Scale bars  = 10 µm.

NTPD2 was also absent from the urothelium (Fig. 4a) but was distributed differentially in the lamina propria (Fig. 4b). The merged images in Fig. 4a and 4b show a region immediately subjacent to urothelium which is actin positive but NTPD2-negative (see asterisks). In the more distal region of the lamina propria, dispersed but interlinked cells with non-uniform morphology are NTPD2-positive. This positive staining pattern extends deep into the detrusor in an organized filamentous pattern which clearly delineates and surrounds smooth muscle bundles (Fig. 4c). These cells exhibit narrow elongated and branched cell processes. We therefore explored the possibility that NTPD2 positive cells were fibroblasts, myofibroblasts or neuronal in origin by co-staining with antibodies for fibroblast-specific protein-1 (FSP1; Fig. 5a), α-smooth muscle actin (αSMA; Fig. 5b) and calcitonin gene related peptide (CGRP; Fig. 5c) respectively. The merged images shown in Fig. 5 clearly illustrate that NTPD2 does not colocalize with any of these three cell markers. FSP1- and αSMA-positive cells were predominantly found in the region of the lamina propria proximate to the urothelium. αSMA staining also indicates blood vessels within the lamina propria (Fig. 5b). Coimmunostaining of neurons revealed that NTPD2 expressing cells are distinct, however in Fig. 5c it can be seen that there is a close association between a well defined neuron and surrounding NTPD2 positive cells. Endothelia did not express NTPD2, in contrast to the expression pattern seen for NTPD1 (Fig. 3b).

**Figure 6:**
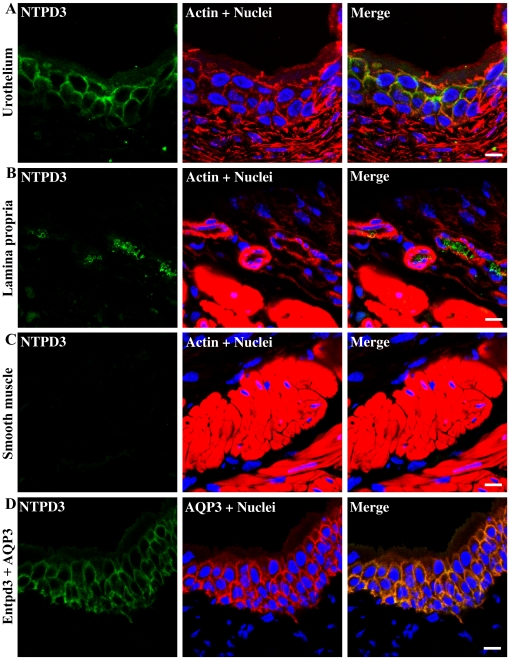
Immunolocalization of NTPD3 in different regions of the bladder. Cryosections of mouse bladders were labeled with antibodies to NTPD3 (green), rhodamine phalloidin to label the actin cytoskeleton (red) and Topro-3 to label nuclei (blue). Color merged panels are shown on the right. A) NTPD3 staining at the level of the urothelium, B) NTPD3 staining at the level of the lamina propria, C) NTPD3 staining at the level of the detrusor smooth muscle, D) NTPD3 staining with a different antibody from (A) at the level of the urothelium. Aquaporin 3 (AQP3), a marker of intermediate and basal cell plasma membranes colocalizes with NTPD3. White scale bars  = 10 µm.

The urothelium is a major source of ATP released in bladder [1]–[4] therefore we were interested to know if any of the NTPDases were expressed by these cells. Fig. 6 shows that NTPD3 is specifically expressed in the urothelium and is differentially localized to the plasma membranes of intermediate and basal cells (Fig. 6a and 6d). The presence of lateral actin staining and corresponding tight junctions in the superficial umbrella cells can be seen in the top middle panel (Fig. 6a). However, there is little evidence for colocalization of NTPD3 at lateral borders of the umbrella cells, indicating that the superficial cells of the urothelium are unlikely to express this enzyme. Antibody staining within the lamina propria is localized to cells within blood vessels and detrusor shows no evidence for NTPD3. A different primary antibody to NTPD3 confirmed the intermediate and basal cell distribution of NTPD3 (Fig. 6d) by precise colocalization with aquaporin 3, a marker of these cell membranes.

**Figure 7:**
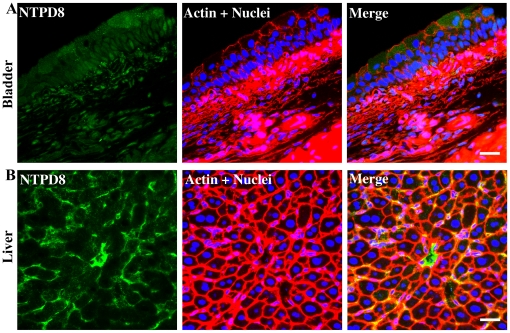
Immunolocalization of NTPD8 in bladder and liver. Cryosections of mouse bladders and livers were labeled with antibodies to NTPD8 (green), rhodamine phalloidin to label the actin cytoskeleton (red) and Topro-3 to label nuclei (blue). Color merged panels are shown on the right. A) NTPD8 staining of bladder, B) NTPD8 staining of liver. White scale bars  = 25 µm.

NTPD8 immunostaining of bladder showed a diffuse relatively non-differentiated signal throughout several regions and there was little evidence for a concentration at cell boundaries (Fig. 7a). There is however a suggestion from the merged images that NTPD8 may be present in the superficial cells of the urothelium but the lack of clear membrane localization for this surface enzyme requires caution in interpretation. To demonstrate the efficacy of the antibody, liver sections were immunostained as a positive control (Fig. 7b). Mouse liver showed specific and higher intensity staining patterns with appropriate cell border localization to canaliculi. A different primary antibody to NTPD8 was also tried but gave identical staining patterns on both bladder and liver sections (not shown). We conclude that expression of NTPD8 may be low and that antibody staining is not sufficiently sensitive to define its location with certainty. RT-PCR (Fig. 1) supports this conclusion with NTPD8 signal lower than for other family members.

**Figure 8:**
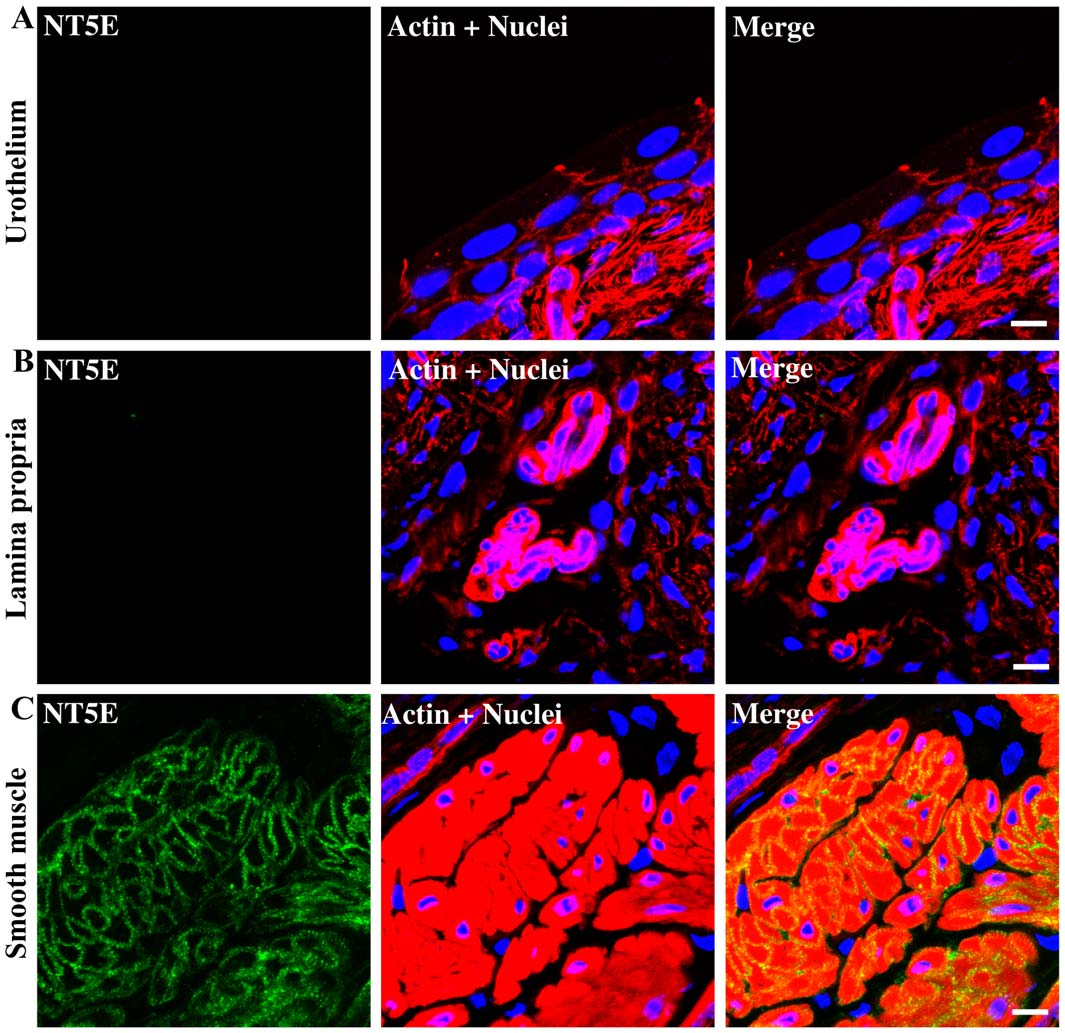
Immunolocalization of NT5E in different regions of the bladder. Cryosections of mouse bladders were labeled with antibodies to NT5E (green), rhodamine phalloidin to label the actin cytoskeleton (red) and Topro-3 to label nuclei (blue). Color merged panels are shown on the right. A) NT5E staining at the level of the urothelium, B) NT5E staining at the level of the lamina propria, C) NT5E staining at the level of the detrusor smooth muscle. White scale bars  = 10 µm.

NT5E which is responsible for the conversion of AMP to adenosine was clearly absent from urothelium (Fig. 8a) and from lamina propria (Fig. 8b) but was present throughout detrusor smooth muscle in a pattern very similar to that seen for NTPD1 (Fig. 8c).

## Discussion

The importance of purinergic signaling for urinary bladder function has become clear, with a broad spectrum of bladder pathologies now known to exhibit aberrant purinergic metabolism. ATP release from the urothelium is significantly elevated in aging [31], interstitial cystitis [10]–[11], in spinal cord injury [15], during inflammation [14] and in syndromes of detrusor overactivity resulting in urgency and/or incontinence [32]. Furthermore overactive bladder has been shown to broadly downregulate the expression of P2X receptors in detrusor [13], [17] while conversely P2X_3_ was upregulated in sensory nerve fibers from patients with neurogenic detrusor overactivity [33]. P2X_3_ is also upregulated in a model of outlet obstruction in rats [18] while human patients with outlet obstruction had elevated P2X_1_ [19] and P2X_2_ [34] receptors in their bladder smooth muscle. P2X_2_/X_3_ are also elevated in urothelium of patients with interstitial cystitis [35]–[36]. Furthermore, visceral pain originating from tube and sac-like organs is now thought to be critically dependent on ATP signaling between epithelia and adjacent sensory neurons [37]-[41]. Therefore painful bladder syndromes of mysterious etiology might occur through mechanisms in which nucleotide signaling is dysregulated or accentuated.

The existence of ATP/ADP degrading enzymes on the surface of cells had been recognized for decades, but molecular identification of the first member of the NTPDase family (NTPDase 1) was not elucidated until the mid-1990s [21]. It is now understood that these enzymes modulate purinergic signaling through effects on ligand availability to P1 and P2 receptors in virtually every tissue of the body and have been shown to play important functional roles in vasculature and the immune and nervous systems [21]. The experiments presented here, are therefore intended to define the expression and localization patterns of NTPDs. We believe this is the first systematic attempt to catalog and describe the location of ectonucleotidases within the mammalian urinary bladder.

We successfully amplified specific mRNA for all eight members of the NTPD family as well as for NT5E, thus confirming the likely importance of modulating nucleotide concentrations within bladder tissue elements (Fig. 1).

**Figure 9:**
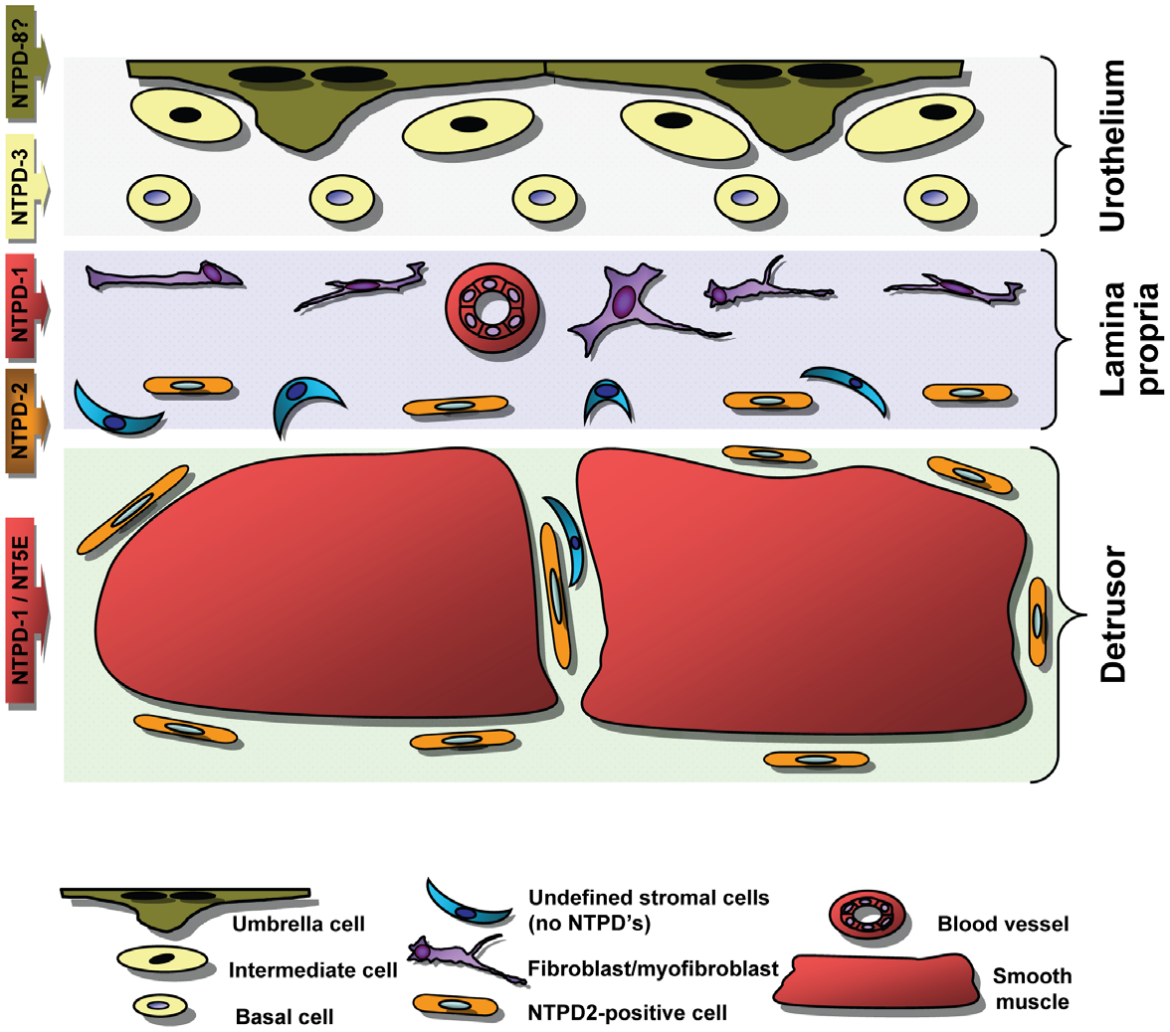
Model of urinary bladder showing differential distribution of nucleotidases. Three tissue layers of the bladder are shown with the cells we have identified as expressing nucleotidases. For the sake of clarity, afferent and efferent neurons have not been included. Different cell types are color coded with the banner arrows at left indicating which colored cells express which nucleotidases. Within the lamina propria, FSP1-positive fibroblasts and α-SMA-positive myofibroblasts localize to the suburothelial region (purple cells) and appear absent or rare closer to the detrusor smooth muscle. The region of the lamina propria adjacent to the smooth muscle is rich in NTPD2-positive cells. They also surround and intercalate between muscle bundles. NTPD8 is indicated in umbrella cells with a question mark to reflect a degree of uncertainty and endothelium of blood vessels are labeled with NTPD1.

Our goal in this study was to characterize the distribution of nucleotide-hydrolyzing enzymes which could modulate the signaling of secreted ATP/UTP. Therefore we focused in more detail on the four cell surface localized enzymes known to specifically catabolize extracellular ATP (NTPD1, -2, -3 and -8) as well as NT5E. Western blotting confirmed that all five were expressed in bladder, but using immunofluorescence we were only able to unequivocally confirm the localization of four, since NTPD8 exhibited low expression levels. Figure 9 schematically illustrates our findings with each enzyme specifically expressed by particular cell types.

NTPD1 is the major ectonucleotidase responsible for degrading ATP within the vasculature and our data clearly show that it is prominently expressed in endothelial cells within bladder. It has been shown to play a key role in hemostasis and thrombosis with complex effects on platelet aggregation [42]. It is likely therefore that its presence in vascular elements within the bladder is not specific to this tissue.

The presence of NTPD1 and NT5E in the cell membranes of smooth muscle cells suggests important functional roles related to muscle contraction and relaxation during the voiding cycle. Indeed, concerted actions are probable given what is known of urinary bladder smooth muscle physiology. To initiate voiding, parasympathetic nerves release ATP to stimulate bladder smooth muscle contraction through P2X_1_ receptors [6]. NTPD1, also present on these membranes, has approximately equal affinities for ATP and ADP [25] and is therefore able to rapidly catalyze the production of AMP. Following the contractile phase of voiding, NTPD1 and NT5E acting coordinately could rapidly convert ATP to adenosine in order to not only effect cessation of P2X_1_-mediated muscle contraction, but to facilitate muscle relaxation through A2b receptors. Relaxation is clearly a prerequisite for accommodating the next filling cycle. Support for this hypothesis comes from studies showing that adenosine receptor, A_2b_ is abundantly expressed in detrusor [43], and further, that adenosine inhibits detrusor contraction elicited through carbachol, electrical field stimulation, acetylcholine and potassium [44]–[46]. This model suggests that ATP is responsible (in part) not only for the contractile phase but also via NTPD1/NT5E activity, the relaxant phase of the micturition cycle and could therefore be considered a ‘dual effector’.

NTPD2 shows an interesting partial distribution in lamina propria, being present in the deeper layer adjacent to the detrusor (illustrated schematically in Fig. 9). Within detrusor, NTPD2-positive cells circumferentially surround and are in close proximity to smooth muscle bundles. Our data show clearly these cells are unlikely to be typical fibroblasts or nerve fibers. In fact based on their branched morphology, network patterning, location around muscle bundles and their intimate association with neurons (Fig. 5c), we considered the possibility that they might be interstitial cells of Cajal (ICC). Despite multiple attempts with different antibodies we were unable to immunostain for the ICC marker, c-kit. c-kit positive ICC, or pacemaker cells, have been convincingly demonstrated in bladders from a number of species including human [47]–[48], dog [49], pig [50], and guinea pig [51]–[52], however studies on bladders of mice are mixed. Lagou *et al.* demonstrated ICC in mouse bladder by morphology and by pharmacological stimulation, however ICC were c-kit negative [53]. Likewise, Pezzone *et al.* also failed to detect c-kit in mouse bladder despite finding c-kit positive cells in the ureter [54]. In contrast to this McCloskey *et al.* were able to show c-kit positive cells in mouse bladder [55]. Despite our inability to colocalize c-kit with NTPD2, there remains a strong circumstantial case for considering that these cells may be ICC.

There are other possibilities also. They may represent a subgroup of myofibroblasts which do not express αSMA. Cells matching this description were identified as myofibroblasts by Liu *et al* [56]. Dranoff *et al.* noted that NTPD2 was present in a novel compartment of fibroblasts in liver, namely portal fibroblasts which surround intrahepatic bile ducts [57]. In another study, myofibroblasts were found to exhibit close contacts with nerve varicosities in electron micrographs and the authors speculated that myofibroblasts and their attached axonal varicosities could collectively function as bladder stretch receptors [58]. Our data show similar intimate contacts between NTPD2-positive cells and neurons. Since this ectoenzyme is highly specific for ATP over ADP (hydrolysis ratio of 1∶0.05–1∶0.3) it's primary function is likely to be in attenuating ATP signaling [25] and generating ADP/UDP ligands for stimulating P2Y receptors in a cellular compartment that lies close to and surrounds the detrusor. It is possible given not only its proximity to smooth muscle but also to nerve fibers that it may play an important role in degrading ATP released as a neurotransmitter from efferent neurons. Fig. 9 shows the cross-boundary distribution of NTPD2-positive cells in both lamina propria and between muscle bundles in the detrusor.

NTPD3 is uniquely localized to the urothelium and interestingly does not appear to present in umbrella cells but occurs in cell membranes of the intermediate and basal cell layers. This explains the observed difference in the kinetics of ATP hydrolysis on the luminal and serosal surfaces [2]. Urothelial stretch-induced ATP secretion lumenally, is likely to have autocrine signaling effects on the apical membranes of umbrella cells while basal release suggests paracrine effects serosally. A primary signaling target for the urothelium is afferent neurons which are closely apposed to and penetrate between basal cells of the lamina propria and even urothelium. The signaling pathways activated by these interactions may influence such diverse phenomena as intracellular Ca^2+^ signaling action potentials and exocytosis of other potent mediators like nitric oxide, acetylcholine and prostaglandins [5]. NTPD3 likely modulates the strength or duration of this stimulus on the cells in which it is expressed i.e. urothelium. Indeed if the primary target for basally secreted ATP is cells in the stroma or detrusor, its role might be to limit the potential for self-stimulation. It is noteworthy that none of the NTPDs were present on the luminal surface of umbrella cells which supports the finding that ATP released apically from urothelium is likely to be long-lived until its expulsion with voided urine.

The location of NTPD8 was uncertain since antibody staining was diffuse and present in several regions. There was however a suggestion that it may be concentrated within umbrella cells. Given the strong expression of this protein within hepatic canaliculus of liver, an epithelial expression pattern in bladder is entirely reasonable. It is however, present at lower levels in the bladder than in the liver.

Figure 9 presents schematically, a simplified overview of our findings, with the bladder structurally divided into three distinct strata; the urothelium, lamina propria and detrusor smooth muscle. NTPD8 and NTPD3 are present exclusively in the epithelium with NTPD3 restricted to the suburothelium. Within the connective tissue elements of the lamina propria, NTPD1 may be found in blood vessels while NTPD2 occurs in a specific subset of cells which may be ICC. Further work will be necessary to confirm if this is true. These cells lie proximal to the smooth muscle and surround muscle bundles, but are not in the smooth muscle itself. NTPD1 and NT5E however, are richly expressed within the smooth muscle suggesting a functionally important relationship.

This expression and localization study provides important novel information about the signature of nucleotidases in mammalian bladder. Knowledge of their tissue-specific distribution will allow the design of rational functional studies to test the contribution of each to normal micturition. For example the use of Cre-lox technology to generate conditional knockouts in specific cell types e.g. urothelium [59], can now be considered for these enzymes. Altered purinergic signaling occurs frequently in bladder disease and at present the involvement of ectonucleotidases in disease processes is completely unknown. This study sets the stage for further investigations of their role in both physiology and pathophysiology. Furthermore, these ectoenzymes may one day offer tempting therapeutic targets for conditions such as overactive bladder or painful bladder syndrome.

## References

[1] Kumar V, Chapple CC, Chess-Williams R (2004). Characteristics of adenosine triphosphate [corrected] release from porcine and human normal bladder.. J Urol.

[2] Lewis SA, Lewis JR (2006). Kinetics of urothelial ATP release.. Am J Physiol Renal Physiol.

[3] Munoz A, Gangitano DA, Smith CP, Boone TB, Somogyi GT (2010). Removal of urothelium affects bladder contractility and release of ATP but not release of NO in rat urinary bladder.. BMC Urol.

[4] Ferguson DR, Kennedy I, Burton TJ (1997). ATP is released from rabbit urinary bladder epithelial cells by hydrostatic pressure changes—a possible sensory mechanism?. J Physiol.

[5] Apodaca G, Balestreire E, Birder LA (2007). The uroepithelial-associated sensory web.. Kidney Int.

[6] Heppner TJ, Werner ME, Nausch B, Vial C, Evans RJ (2009). Nerve-evoked purinergic signalling suppresses action potentials, Ca2+ flashes and contractility evoked by muscarinic receptor activation in mouse urinary bladder smooth muscle.. J Physiol.

[7] Lee HY, Bardini M, Burnstock G (2000). Distribution of P2X receptors in the urinary bladder and the ureter of the rat.. J Urol.

[8] Vlaskovska M, Kasakov L, Rong W, Bodin P, Bardini M (2001). P2X3 knock-out mice reveal a major sensory role for urothelially released ATP.. J Neurosci.

[9] Chopra B, Gever J, Barrick SR, Hanna-Mitchell AT, Beckel JM (2008). Expression and function of rat urothelial P2Y receptors.. Am J Physiol Renal Physiol.

[10] Sun Y, Chai TC (2006). Augmented extracellular ATP signaling in bladder urothelial cells from patients with interstitial cystitis.. Am J Physiol Cell Physiol.

[11] Sun Y, Chai TC (2002). Effects of dimethyl sulphoxide and heparin on stretch-activated ATP release by bladder urothelial cells from patients with interstitial cystitis.. BJU Int.

[12] Birder LA, Barrick SR, Roppolo JR, Kanai AJ, de Groat WC (2003). Feline interstitial cystitis results in mechanical hypersensitivity and altered ATP release from bladder urothelium.. Am J Physiol Renal Physiol.

[13] Moore KH, Ray FR, Barden JA (2001). Loss of purinergic P2X(3) and P2X(5) receptor innervation in human detrusor from adults with urge incontinence.. J Neurosci.

[14] Smith CP, Vemulakonda VM, Kiss S, Boone TB, Somogyi GT (2005). Enhanced ATP release from rat bladder urothelium during chronic bladder inflammation: effect of botulinum toxin A.. Neurochem Int.

[15] Smith CP, Gangitano DA, Munoz A, Salas NA, Boone TB (2008). Botulinum toxin type A normalizes alterations in urothelial ATP and NO release induced by chronic spinal cord injury.. Neurochem Int.

[16] Kumar V, Chapple CR, Rosario D, Tophill PR, Chess-Williams R (2009). In Vitro Release of Adenosine Triphosphate from the Urothelium of Human Bladders with Detrusor Overactivity, Both Neurogenic and Idiopathic..

[17] Ray FR, Moore KH, Hansen MA, Barden JA (2003). Loss of purinergic P2X receptor innervation in human detrusor and subepithelium from adults with sensory urgency.. Cell Tissue Res.

[18] Kim JC, Yoo JS, Park EY, Hong SH, Seo SI (2008). Muscarinic and purinergic receptor expression in the urothelium of rats with detrusor overactivity induced by bladder outlet obstruction.. BJU Int.

[19] O'Reilly BA, Kosaka AH, Chang TK, Ford AP, Popert R (2001). A quantitative analysis of purinoceptor expression in the bladders of patients with symptomatic outlet obstruction.. BJU Int.

[20] Chua WC, Liu L, Mansfield KJ, Vaux KJ, Moore KH (2007). Age-related changes of P2X(1) receptor mRNA in the bladder detrusor from men with and without bladder outlet obstruction.. Exp Gerontol.

[21] Robson SC, Sevigny J, Zimmermann H (2006). The E-NTPDase family of ectonucleotidases: Structure function relationships and pathophysiological significance.. Purinergic Signal.

[22] Sevigny J, Sundberg C, Braun N, Guckelberger O, Csizmadia E (2002). Differential catalytic properties and vascular topography of murine nucleoside triphosphate diphosphohydrolase 1 (NTPDase1) and NTPDase2 have implications for thromboregulation.. Blood.

[23] Sakagami H, Aoki J, Natori Y, Nishikawa K, Kakehi Y (2005). Biochemical and molecular characterization of a novel choline-specific glycerophosphodiester phosphodiesterase belonging to the nucleotide pyrophosphatase/phosphodiesterase family.. J Biol Chem.

[24] Gijsbers R, Aoki J, Arai H, Bollen M (2003). The hydrolysis of lysophospholipids and nucleotides by autotaxin (NPP2) involves a single catalytic site.. FEBS Lett.

[25] Vorhoff T, Zimmermann H, Pelletier J, Sevigny J, Braun N (2005). Cloning and characterization of the ecto-nucleotidase NTPDase3 from rat brain: Predicted secondary structure and relation to other members of the E-NTPDase family and actin.. Purinergic Signal.

[26] Stefan C, Jansen S, Bollen M (2005). NPP-type ectophosphodiesterases: unity in diversity.. Trends Biochem Sci.

[27] Stefan C, Jansen S, Bollen M (2006). Modulation of purinergic signaling by NPP-type ectophosphodiesterases.. Purinergic Signal.

[28] Shirley DG, Vekaria RM, Sevigny J (2009). Ectonucleotidases in the kidney.. Purinergic Signal.

[29] Canani LH, Ng DP, Smiles A, Rogus JJ, Warram JH (2002). Polymorphism in ecto-nucleotide pyrophosphatase/phosphodiesterase 1 gene (ENPP1/PC-1) and early development of advanced diabetic nephropathy in type 1 diabetes.. Diabetes.

[30] Yu W, Zacharia LC, Jackson EK, Apodaca G (2006). Adenosine receptor expression and function in bladder uroepithelium.. Am J Physiol Cell Physiol.

[31] Yoshida M, Miyamae K, Iwashita H, Otani M, Inadome A (2004). Management of detrusor dysfunction in the elderly: changes in acetylcholine and adenosine triphosphate release during aging.. Urology.

[32] Kumar V, Chapple CR, Rosario D, Tophill PR, Chess-Williams R (2010). In vitro release of adenosine triphosphate from the urothelium of human bladders with detrusor overactivity, both neurogenic and idiopathic.. Eur Urol.

[33] Brady CM, Apostolidis A, Yiangou Y, Baecker PA, Ford AP (2004). P2X3-immunoreactive nerve fibres in neurogenic detrusor overactivity and the effect of intravesical resiniferatoxin.. Eur Urol.

[34] O'Reilly BA, Kosaka AH, Knight GF, Chang TK, Ford AP (2002). P2X receptors and their role in female idiopathic detrusor instability.. J Urol.

[35] Sun Y, Chai TC (2004). Up-regulation of P2X3 receptor during stretch of bladder urothelial cells from patients with interstitial cystitis.. J Urol.

[36] Tempest HV, Dixon AK, Turner WH, Elneil S, Sellers LA (2004). P2X and P2X receptor expression in human bladder urothelium and changes in interstitial cystitis.. BJU Int.

[37] Burnstock G (2009). Purinergic mechanosensory transduction and visceral pain.. Mol Pain.

[38] Burnstock G (2009). Purinergic receptors and pain.. Curr Pharm Des.

[39] Burnstock G (2001). Purine-mediated signalling in pain and visceral perception.. Trends Pharmacol Sci.

[40] Cockayne DA, Hamilton SG, Zhu QM, Dunn PM, Zhong Y (2000). Urinary bladder hyporeflexia and reduced pain-related behaviour in P2X3-deficient mice.. Nature.

[41] Burnstock G (1996). A unifying purinergic hypothesis for the initiation of pain.. Lancet.

[42] Enjyoji K, Sevigny J, Lin Y, Frenette PS, Christie PD (1999). Targeted disruption of cd39/ATP diphosphohydrolase results in disordered hemostasis and thromboregulation.. Nat Med.

[43] Stehle JH, Rivkees SA, Lee JJ, Weaver DR, Deeds JD (1992). Molecular cloning and expression of the cDNA for a novel A2-adenosine receptor subtype.. Mol Endocrinol.

[44] Brown C, Burnstock G, Cocks T (1979). Effects of adenosine 5′-triphosphate (ATP) and beta-gamma-methylene ATP on the rat urinary bladder.. Br J Pharmacol.

[45] King JA, Huddart H, Staff WG (1997). Purinergic modulation of rat urinary bladder detrusor smooth muscle.. Gen Pharmacol.

[46] Nicholls J, Hourani SM, Kitchen I (1992). Characterization of P1-purinoceptors on rat duodenum and urinary bladder.. Br J Pharmacol.

[47] Johnston L, Woolsey S, Cunningham RM, O'Kane H, Duggan B (2010). Morphological expression of KIT positive interstitial cells of Cajal in human bladder.. J Urol.

[48] Shafik A, El-Sibai O, Shafik AA, Shafik I (2004). Identification of interstitial cells of Cajal in human urinary bladder: concept of vesical pacemaker.. Urology.

[49] Arrighi S, Bosi G, Groppetti D, Cremonesi F (2010). Identification of C-kit-positive interstitial cells in the dog lower urinary tract and relationship with smooth muscle and nerves..

[50] Metzger R, Neugebauer A, Rolle U, Bohlig L, Till H (2008). C-Kit receptor (CD117) in the porcine urinary tract.. Pediatr Surg Int.

[51] Davidson RA, McCloskey KD (2005). Morphology and localization of interstitial cells in the guinea pig bladder: structural relationships with smooth muscle and neurons.. J Urol.

[52] McCloskey KD, Gurney AM (2002). Kit positive cells in the guinea pig bladder.. J Urol.

[53] Lagou M, De Vente J, Kirkwood TB, Hedlund P, Andersson KE (2006). Location of interstitial cells and neurotransmitters in the mouse bladder.. BJU Int.

[54] Pezzone MA, Watkins SC, Alber SM, King WE, de Groat WC (2003). Identification of c-kit-positive cells in the mouse ureter: the interstitial cells of Cajal of the urinary tract.. Am J Physiol Renal Physiol.

[55] McCloskey KD, Anderson UA, Davidson RA, Bayguinov YR, Sanders KM (2009). Comparison of mechanical and electrical activity and interstitial cells of Cajal in urinary bladders from wild-type and W/Wv mice.. Br J Pharmacol.

[56] Liu F, Takahashi N, Yamaguchi O (2009). Expression of P2X3 purinoceptors in suburothelial myofibroblasts of the normal human urinary bladder.. Int J Urol.

[57] Dranoff JA, Kruglov EA, Robson SC, Braun N, Zimmermann H (2002). The ecto-nucleoside triphosphate diphosphohydrolase NTPDase2/CD39L1 is expressed in a novel functional compartment within the liver.. Hepatology.

[58] Wiseman OJ, Fowler CJ, Landon DN (2003). The role of the human bladder lamina propria myofibroblast.. BJU Int.

[59] Zhou H, Liu Y, He F, Mo L, Sun TT (2010). Temporally and spatially controllable gene expression and knockout in mouse urothelium.. Am J Physiol Renal Physiol.

